# Metal-Induced Oxidative Stress and Plant Mitochondria

**DOI:** 10.3390/ijms12106894

**Published:** 2011-10-18

**Authors:** Els Keunen, Tony Remans, Sacha Bohler, Jaco Vangronsveld, Ann Cuypers

**Affiliations:** Centre for Environmental Sciences, Hasselt University, Agoralaan Building D, B-3590 Diepenbeek, Belgium; E-Mails: els.keunen@uhasselt.be (E.K.); tony.remans@uhasselt.be (T.R.); sacha.bohler@uhasselt.be (S.B.); jaco.vangronsveld@uhasselt.be (J.V.)

**Keywords:** toxic metals, oxidative stress, reactive oxygen species (ROS), plant mitochondria, oxidative damage, signaling

## Abstract

A general status of oxidative stress in plants caused by exposure to elevated metal concentrations in the environment coincides with a constraint on mitochondrial electron transport, which enhances ROS accumulation at the mitochondrial level. As mitochondria are suggested to be involved in redox signaling under environmental stress conditions, mitochondrial ROS can initiate a signaling cascade mediating the overall stress response, *i.e.*, damage *versus* adaptation. This review highlights our current understanding of metal-induced responses in plants, with focus on the production and detoxification of mitochondrial ROS. In addition, the potential involvement of retrograde signaling in these processes will be discussed.

## 1. Introduction

Worldwide, metal industry and agricultural use of metal-containing fertilizers and pesticides have contributed significantly to metal pollution. Resulting concentrations of toxic metals in the environment often exceed those from natural sources [1]. Metals such as copper (Cu), iron (Fe), nickel (Ni) and zinc (Zn) are essential for functioning of physiological and biochemical processes and, consequently, for normal growth and development of organisms [2,3]. However, elements such as aluminium (Al), cadmium (Cd) and lead (Pb) are considered to be non-essential and generate toxic responses even at low exposure concentrations. An excess of toxic metals commonly has a negative impact on physiological and biochemical processes in organisms, resulting in major risks to the environment and also for human health. Numerous *in vitro* and *in vivo* population-based studies have demonstrated the risks of enhanced metal exposure for human health [4–7]. If toxic metals accumulate in crop plants, the uptake of potentially hazardous elements via the food chain tremendously increases in humans [8]. A better understanding of metal-induced molecular, cellular and physiological responses in plants will therefore contribute to the development or adjustment of strategies to alleviate metal-associated risks for human health.

Plants growing on metal-enriched soils suffer from decreased growth and performance, both restricting crop yield. At the molecular level, oxidative stress is widely studied as a key sign of plant stress. This process is commonly described as an imbalance between reactive oxygen species (ROS) and antioxidants in favor of the former. Sharma and Dietz [9] recently summarized the intense relationship between metal toxicity, redox homeostasis and antioxidant capacity in plants. Depending on the chemical properties and behavior of metals in biological systems, their toxicity is attributed to either one of the following mechanisms: (1) interference with functional sites in proteins; (2) displacement of essential elements, thereby disturbing enzymatic functions; or (3) enhanced ROS production [9]. Redox-active metals such as Cu and Fe directly induce ROS production through Fenton and Haber-Weiss reactions [10,11]. Non-redox-active metals (Cd, Pb and Zn) however only enhance ROS production via indirect mechanisms such as inhibiting enzymes functioning in the cellular antioxidative defense network [10]. While an enzymatic oxidative burst mediated by NADPH oxidases is well studied under conditions of pathogen attack, a clear role for these ROS producing enzymes after metal application is demonstrated in various studies [12,13]. In addition to enzymatic pathways, excess metals increase ROS production in subcellular organelles such as peroxisomes, chloroplasts and mitochondria, which together constitute the predominant sources of ROS production in plants. Their highly oxidizing nature and the presence of electron transport chains in chloroplasts and mitochondria makes both organelles a preferential site for metal-induced ROS production [11,14]. Chloroplasts are intensively studied in the light of photosynthesis and its accompanying ROS production under metal stress conditions [15]. However, research has extended to plant mitochondria in recent years [9,16]. A clear relationship was demonstrated between metal stress, redox homeostasis and antioxidant metabolism at the cytosolar and organellar level [9]. As mitochondria are key players in cellular redox homeostasis and signaling [16], this review focuses on ROS production and antioxidative defense mechanisms in mitochondria and how these processes are affected by metal exposure. The resulting oxidative challenge in mitochondria further implicates downstream signaling responses via ROS and/or other signaling intermediates, which will be discussed in the light of metal stress in this review.

## 2. Metal-Induced ROS Production: Interplay between Cytosol and Mitochondria

**Figure 1:**
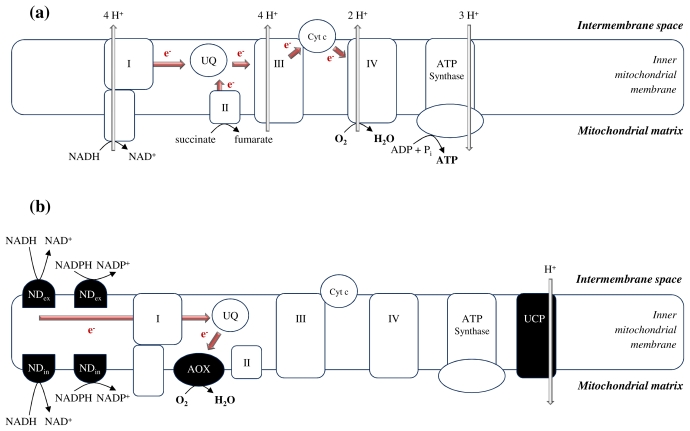
Simplified overview of the components involved in conventional and alternative reactions of mitochondrial electron transport and oxidative phosphorylation in plants. (**a**) In the “standard” cytochrome pathway, electrons pass from respiratory complexes I (NADH dehydrogenase) and II (succinate dehydrogenase) to the electron carrier ubiquinone (UQ). Via complex III (ubiquinol-cytochrome *bc*_1_ reductase) and cytochrome c (cyt c), O_2_ is ultimately reduced to H_2_O at the level of complex IV (cytochrome c oxidase). The ATP synthase complex catalyzes the formation of ATP in the mitochondrial matrix driven by the proton gradient resulting from electron transfer; (**b**) In addition, plant mitochondria contain an alternative pathway consisting of a non-proton-pumping alternative oxidase (AOX) as well as alternative NAD(P)H dehydrogenases (NDs) on either the external (ND_ex_) or internal (ND_in_) side of the inner mitochondrial membrane. Electrons are passed from the alternative NDs to ubiquinone and directly to AOX reducing O_2_ to H_2_O. Uncoupling proteins (UCPs) are able to dissipate the proton electrochemical gradient over the inner membrane created by the transfer of electrons, thereby acting as an alternative path to mitochondrial oxidative phosphorylation.

**Figure 2:**
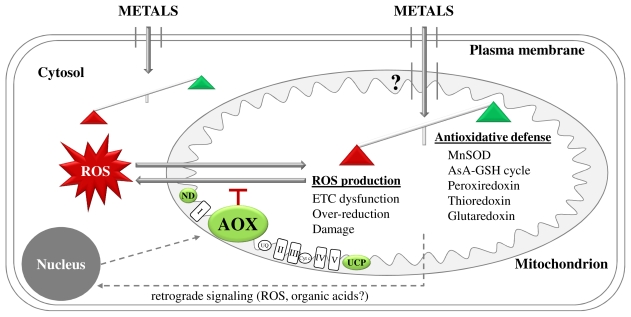
Schematic overview of metal-induced responses in plant cells focusing on mitochondrial effects. Metal exposure has shown to cause mitochondrial electron transport chain (ETC) dysfunction and over-reduction, thereby increasing mitochondrial ROS production. However, more research is needed to determine whether this is the direct consequence of metals entering the mitochondria, since cytosolar ROS production cannot be excluded in the light of metal stress and can influence mitochondrial responses. As they are able to cross cellular membranes, ROS serve signaling functions outside the mitochondria (dashed line) and can induce retrograde signaling to the nucleus, which could also be regulated via organic acids. As AOX is able to reduce mitochondrial ROS production, modulate programmed cell death (PCD) and tricarboxylic acid (TCA) cycle activity, this enzyme is suggested to play a key role in metal-induced responses in plant mitochondria.

Mitochondria are the principal organelles performing plant aerobic respiration. In this process, organic acids are oxidized to CO_2_ and H_2_O in the tricarboxylic acid (TCA) cycle in the mitochondrial matrix, thereby fuelling electrons from reducing NADH (and FADH_2_) equivalents to O_2_ via the respiratory electron transport chain (ETC) present in the inner mitochondrial membrane (Figure 1). This electron transfer process is coupled to the synthesis of energy in the form of ATP in a process termed oxidative phosphorylation [17]. When plants are exposed to toxic metals, apoplastic transport followed by cytosolar uptake and distribution of metals to organelles causes ROS generation due to their redox-active nature or the effects on subcellular metabolism [9]. Van Belleghem *et al*. [18] have shown both cytosolar and vacuolar sequestration of Cd using energy-dispersive X-ray microanalysis (EDXMA) on high-pressure frozen and freeze-substituted tissues of *Arabidopsis* seedlings chronically exposed to 0.1, 1, 5 or 50 μM Cd via the roots. However, no Cd could be detected in mitochondria [18]. As recently reviewed by Nouet *et al*. [19], the proteins involved in metal import into plant mitochondria remain mostly unknown. Therefore, it is important to keep in mind that mitochondrial responses during metal exposure are not necessarily evoked by metals reaching and accumulating in these organelles *per se*. Nevertheless, it is crucial to study the potential interplay between cytosolar and mitochondrial responses to metal stress in plants in order to obtain further insights into a broader cellular picture (Figure 2). To unravel metal-induced mitochondrial responses, these organelles should be isolated from different organs of plants exposed to excess metals to obtain full and organelle-specific transcriptome, proteome and/or enzyme activity spectra. In this regard, care has to be taken as the experimental setup will definitely influence the outcome. Metal speciation needs to be considered as it modulates the results when exposing either plants or cell suspension cultures. Moreover, exposing whole plants or cell cultures to metals prior to isolating mitochondria will lead to other—potentially conflicting—results as compared to exposing isolated mitochondria to excess metals. Which setup to choose ultimately depends on the research question, but it is key to interpret all results in a broader context.

### 2.1. The Unique Demands Placed on Plant Mitochondria

In addition to respiration, plant mitochondria play a role in the metabolism of diverse amino acids, vitamins and lipids essential for organellar biogenesis and maintenance. The relative importance of these processes depends on the cell type and developmental stage. Studying the responses of plant mitochondria to metal stress implies the need to fractionate the responses in different plant organs. In leaves, the cellular environment of plant mitochondria is rather distinctive as compared to animal cells due to the presence of photosynthesis-derived O_2_ and carbohydrates. In plant roots however, a different cellular environment as compared to the leaves will definitely influence the response of mitochondria to excess metals [16,17]. It is noteworthy to mention that plant mitochondria differ from their animal counterparts in that the former function in distinct processes, such as photorespiration, and that they possess unique components (alternative oxidase (AOX), alternative NAD(P)H dehydrogenases (NDs) and uncoupling proteins (UCPs) (Figure 1b) [16,17,20]. Electrons can pass through either the “standard” cytochrome pathway to complex IV (cytochrome c oxidase) (Figure 1a) or through the alternative pathway to the cyanide-insensitive AOX (Figure 1b). Although this alternative route does not contribute to ATP synthesis, AOX is currently considered both a target and regulator of stress responses in plants (Sections 3.1 and 4.3) [21,22].

### 2.2. Metal Exposure Increases ROS Generation in Plant Mitochondria

Under normal conditions, the mitochondrial ETC and ATP synthesis are tightly coupled. However, plants suffering from biotic or abiotic stress often show an over-reduction of electron carriers such as ubiquinone, causing electron leakage from the system. These electrons possess a sufficient amount of free energy to directly reduce molecular O_2_, with increased production of ROS such as superoxide (O_2_°*^−^*) and hydrogen peroxide (H_2_O_2_) as unavoidable byproducts of aerobic metabolism [17,21,23–26].

The known sites of ROS production in the mitochondrial ETC are complexes I and III (for reviews, see [21,23]), where O_2_°*^−^* is formed and subsequently dismutated to H_2_O_2_. The uncharged H_2_O_2_ molecule is able to penetrate membranes and has a longer half-life as compared to O_2_°*^−^*. Both properties make H_2_O_2_ an ideal candidate for ROS signaling from mitochondria to other organelles [17]. However, H_2_O_2_ can also react with reduced Fe*^2+^* and Cu*^+^* in the mitochondrion itself to produce highly toxic hydroxyl radicals and cause oxidative damage to mitochondrial proteins, lipids and DNA [21,25,27].

In mitochondria, transition metals such as Cu, Fe and Zn are crucial for a proper functioning of several enzymes involved in the TCA cycle, electron transport, synthesis of ATP and antioxidative defense [19,27]. However, the presence of free (redox-active) metal cations can initiate or propagate oxidative stress (Table 1). Results of numerous studies investigating plant metal stress responses point toward the generation of oxidative stress and mitochondrial dysfunction as determinants in metal-induced cytotoxicity. In several plant species, metal stress enhances mitochondrial ROS generation mainly by affecting respiratory gas exchange rates [28]. An excess of redox-active metals such as Fe and Cu influences mitochondrial respiration activity (Table 1) [29,30], which could be related to their direct potential to increase mitochondrial ROS production via Fenton and Haber-Weiss reactions. However, also non-redox-active metals negatively affect respiration processes (Table 1). Dixit *et al*. [31] studied the *in vivo* effects of chromium (Cr) in pea root mitochondria and observed an inactivated electron transport and enhanced generation of O_2_*°* *^−^*. The inhibition of root elongation after exposing pea to Al was attributed to metal-induced ROS production, inhibition of respiration and ATP depletion [32]. However, as adenylates show a drastic decrease in response to phosphate deficiency [33], which commonly coincides with Al exposure due to restricted phosphorus uptake [34], the indicative value of the observed ATP depletion is rather low. Instead, one should determine the ATP/ADP ratio to adequately rationalize the effects of metals on the efficiency of the mitochondrial respiratory chain.

Similar to animals [35], the plant mitochondrial ETC is also considered to be an important target of Cd toxicity [36–39]. Heyno *et al*. [36] demonstrated a fast Cd-induced stimulation of ROS generation inside root cells, mainly originating from the mitochondrial ETC. Exposure to Cd impairs proper mitochondrial functioning partly by affecting the organellar redox balance as shown by Smiri *et al*. [39]. Bi *et al*. [37] confirmed ROS production in mitochondria prior to chloroplasts in combination with altered mitochondrial distribution and mobility patterns in Cd-exposed *Arabidopsis thaliana* protoplasts (Table 1).

In addition to ROS, plant mitochondria are also able to produce nitric oxide (NO) under low oxygen levels that affects the activity of mitochondrial ETC and matrix enzymes and transcriptionally upregulates AOX. The role NO plays during oxidative signaling falls out of the scope of this review, but was reviewed in detail elsewhere [25,40]. A potential role for NO during metal stress responses in plants is suggested by the results of De Michele *et al*. [41], who demonstrated the involvement of NO in Cd-induced programmed cell death of *Arabidopsis thaliana* suspension cultures. Arasimowicz**-**Jelonek *et al*. [42] recently discussed the mode of action of NO during Cd stress in plants, with a potential intense relationship between NO and ROS signaling during metal stress as was also shown to occur in plant responses to biotic stress.

## 3. Mechanisms to Control Mitochondrial ROS Production under Metal Stress

Plants contain a dynamic antioxidative defense network to counterbalance the accumulation of ROS, thereby limiting their detrimental effects while still allowing redox signaling throughout the plant [21,25]. During environmental stress conditions such as metal exposure, an integrated antioxidative response in and between different cellular organelles and compartments is required to locally act against ROS production [25,54]. Sweetlove *et al*. [55] studied the impact of oxidative stress induced by H_2_O_2_, menadione (an intracellular O_2_°^−^ generator) or antimycin A (an inhibitor of respiratory complex III) in *Arabidopsis* cells and provided direct evidence for plant mitochondria using an array of enzymes to detoxify ROS (thioredoxin-based redox pathway) or repair oxidative stress-induced damage (protein disulphide isomerase) [55] (Figure 2). Based on the fact that metals induce mitochondrial ROS production and thus oxidative stress, it could be rationalized that similar mechanisms may be involved to counterbalance the accumulation of ROS. In the following paragraphs, the potential mechanisms exploited by plant mitochondria to avoid or detoxify metal-induced ROS will be discussed.

### 3.1. Avoidance of Mitochondrial ROS Production at the ETC Level as a First Line of Defense

Stress-induced over-reduction of ETC components and resulting electron leakage from the system is a principal cause of mitochondrial ROS production. Plant mitochondria contain energy-dissipating systems able to regulate the mitochondrial membrane potential, thereby decreasing mitochondrial ROS production due to ETC over-reduction. However, one must keep in mind that these systems are not able to prevent damage by cytosolar ROS diffusing into the mitochondria [56]. Van Dongen *et al*. [57] recently reviewed the important role of alternative pathways regulating plant respiration. These pathways ensure metabolic adaptation to hypoxia or altered O_2_ availability, which could also indicate their importance during metal stress conditions in plants.

Plant mitochondria contain several proteins present in the vicinity of the “classical” ETC components, which can alleviate the degree of coupling between electron transport and ATP synthesis. They bypass ETC complexes by diverting electrons from the primary cytochrome c pathway, while energy is dissipated as heat. The first enzyme to be discussed in the context of this alternative pathway is AOX, a terminal oxidase accepting electrons directly from ubiquinone and thereby bypassing complexes III and IV (Figure 1b). Since it was first suggested by Purvis and Shewfelt [58], several studies confirmed that AOX prevents mitochondrial oxidative stress (Figure 2). Maxwell *et al*. [59] demonstrated a direct link between functional AOX levels and mitochondrial ROS production in *Arabidopsis* cells, thereby confirming the ability of AOX to reduce mitochondrial ROS levels. Exposure to several metals has been shown to affect the alternative respiratory pathway mediated by AOX at different levels (Table 1). Excess Cu induced AOX at transcriptional and protein levels in sycamore cells [30]. In the protist *Euglena gracilis*, Cd stress led to an increased AOX content and capacity [60]. This was also demonstrated in barley plants, where Cd exposure altered the contribution of the alternative respiratory pathway to total respiration as measured by O_2_ uptake in the presence of the AOX inhibitor salicylhydroxamic acid (SHAM). The authors have shown strong effects of high Cd concentrations on total and alternative respiratory rate and suggested the activated alternative respiration to act as a homeostasis mechanism in Cd-stressed root cells [50]. In addition, Cr exposure enhanced the alternative respiratory rate in *Salvinia* leaves [51]. Pea root tissues exposed to Pb showed a dose-dependent increase in the expression of genes coding for AOX, which was immunologically confirmed [45]. Also, the transcript level of the major AOX isoform in *Arabidopsis* (*AOX1a*) increased in Al-stressed protoplasts in a time-dependent way [44]. From these observations, it is clear that AOX activation could be involved in avoiding metal-induced oxidative stress in plants.

In addition to AOX, plant mitochondria contain alternative NDs able to oxidize cytosolic or matrix NADH/NADPH, thereby bypassing complex I and reducing ubiquinone without pumping protons across the inner membrane (Figure 1b) [61]. Although co-regulated expression patterns for several members of the AOX and alternative ND families were detected under multiple stress conditions affecting mitochondrial respiration [62,63], more research is needed to explore the potential role of alternative NDs during metal stress in plants. Results of our work indicate a possible co-regulation of *AOX* and alternative *ND* transcription in Cd-exposed *Arabidopsis* roots and leaves [64]. This suggests the formation of an abridged but functional alternative respiratory chain with electrons from the alternative NDs passing to AOX via ubiquinone (Figure 1b) under conditions comprising the mitochondrial ETC such as Cd exposure.

Further, mitochondrial UCPs catalyze a proton leak that dissipates the proton electrochemical gradient over the inner mitochondrial membrane, thereby shortcutting the ATP synthase complex and thus oxidative phosphorylation (Figure 1b) [65]. A number of observations suggest a role for UCPs mediating cellular tolerance to oxidative stress [66]. Superoxide [67] and lipid peroxidation products [68] stimulate UCP activity in plant mitochondria. The expression of genes coding for UCP is induced by low temperature conditions [69] and treatment with H_2_O_2_ [70,71]. Overexpressing a gene coding for UCP conferred tolerance to oxidative stress in rice [72] and lack of UCP induced localized oxidative stress in *Arabidopsis* [73]. In durum wheat mitochondria, it was shown that drought stress induced ROS production, which further promoted UCP activity. Thereby, the authors validated a feedback link between stress-induced mitochondrial ROS production and UCP-mediated inhibition of further ROS accumulation [25,74]. As metals induce mitochondrial oxidative stress and ROS production (Section 2.2), a role for UCP is plausible and further research should be conducted in this area. Promising results were published by Yin *et al*. [75], who demonstrated increased lipid peroxidation products shown to activate UCP [68] in Al-stressed tobacco roots. Although both AOX and UCP activity confer dissipation of energy as heat, they respond to different environmental stimuli. Rasmusson *et al*. [76] suggested AOX activity to be involved in the acute response to ETC over-reduction, while UCP could become important during prolonged mitochondrial oxidative stress based on their direct *versus* indirect effect on the transfer of electrons. In addition, the AOX enzyme can be inhibited by lipid peroxidation products, while UCP is stimulated by O_2_°^−^ [63,77], thereby signifying the role both enzymes can play in metal-induced oxidative stress as mentioned above.

### 3.2. Mitochondrial Enzymes and Metabolites Involved in the Detoxification of Mitochondrial ROS

Once formed, O_2_°^−^ radicals are rapidly dismutated to H_2_O_2_ via the manganese superoxide dismutase (MnSOD) enzyme present in the mitochondrial matrix [56,78]. It was shown that transition metals such as Cu, Fe and Zn do not affect MnSOD activity in tobacco cell cultures [79]. However, *MSD* (MnSOD) transcript levels were slightly affected by Cd and Cu exposure in *Arabidopsis thaliana* [52] and the MnSOD activity in pea root mitochondria increased after Pb exposure [46]. These apparent conflicting results (Table 1) can be attributed to the chosen experimental setup. The observed effects can differ when measured on whole plants *versus* cell cultures and depending on the used metal concentrations, which greatly hinders accurate interpretation of the available experimental data. However, a role for MnSOD in the adaptation to Al stress is suggested by experimental data using transgenic plants overexpressing MnSOD. Aluminium-induced inhibition of root growth, lipid peroxidation and callose accumulation—a marker for Al injury—decreased in homozygous transgenic overexpressor plants as compared to wildtypes. In conclusion, the authors suggested an improved resistance to Al toxicity mediated by MnSOD in *Brassica napus* [49].

In order to provide optimal defense against O_2_°^−^-derived H_2_O_2_ production, MnSOD must act in concert with H_2_O_2_-scavenging components. An important system removing H_2_O_2_ is the ascorbate-glutathione cycle comprised of four enzymes (ascorbate peroxidase (APX), dehydroascorbate reductase (DHAR), monodehydroascorbate reductase (MDHAR) and glutathione reductase (GR)) and two metabolites (ascorbate (AsA) and glutathione (GSH)) that are regenerated using NADPH equivalents [80]. Jiménez *et al*. [81] demonstrated the presence of this cycle in plant mitochondria and several cycle enzymes were shown to be dually targeted to mitochondria and chloroplasts [82]. In general, several reports demonstrated that metal stress affects the plant AsA-GSH cycle at both enzyme and metabolite levels (Table 1; Seth *et al*. [14] and references therein) and mitochondria show an interesting link to this cycle as they harbor the last enzyme in the AsA biosynthesis pathway. In this final step, l-galactono-γ-lactone (GL) is converted to AsA by the membrane-bound l-galactono-γ-lactone dehydrogenase (GLDH), which uses cytochrome c as an electron acceptor and thereby donates electrons to the ETC [25,83]. Zhao *et al*. [84] have demonstrated a protective role for GL during Cd stress in winter wheat. Application of this AsA precursor lowered Cd-induced H_2_O_2_ production and increased peroxidase activities [84]. In addition to AsA biosynthesis, it was recently shown that AsA regeneration from dehydroascorbate is also coupled to the plant mitochondrial ETC at the level of complex II, *i.e.*, succinate dehydrogenase [25,85]. As metals strongly affect this complex [50,86], they can also influence the rate of AsA reduction under stress conditions when AsA functions as an important antioxidant.

Plant mitochondria also contain peroxiredoxin, thioredoxin and glutaredoxin enzyme systems capable of scavenging H_2_O_2_. These enzymes became a topic of great interest over the recent years (for a review see [56]). In *Arabidopsis thaliana* cell cultures, Cd application increased the mitochondrial peroxiredoxin content (Table 1) [47]. Finkemeier *et al*. [87] demonstrated a principal role for the mitochondrial peroxiredoxin isoform F (PrxII F) in antioxidant defense and potential redox signaling in plant cells using knockout (KO)-*At*PrxII F *Arabidopsis* seedlings. Under CdCl_2_ exposure and after SHAM-administration, the root growth of KO seedlings was more compromised as compared to wildtype seedlings, thereby signifying the involvement of this mitochondrial peroxiredoxin in Cd detoxification [87]. Gelhaye *et al*. [88] have demonstrated the presence of a mitochondrial thioredoxin isoform in plant mitochondria capable of AOX regulation. This suggests its involvement in metal-induced mitochondrial responses via AOX-mediated modulation of ROS formation. Lastly, Smiri *et al*. [39] observed a Cd-evoked decrease in glutaredoxin activity measured in burst mitochondria extracts from germinating pea seeds. Although these results point towards the possible involvement of peroxiredoxin, thioredoxin and glutaredoxin enzyme systems in mitochondrial stress responses, the exact mechanisms under metal exposure need to be resolved.

## 4. Cellular Acclimation to Metal Exposure in Relation to Plant Mitochondria

Changes in mitochondrial electron transport and/or ROS production can have consequences for all other organelles in the plant cell. Indeed, plant mitochondria have a central position in the cellular carbon and nitrogen metabolism via the TCA cycle and their role in photorespiration [17]. Dutilleul *et al*. [20] have demonstrated this using a *Nicotiana sylvestris* mutant (CMSII) lacking functional complex I. This induces signaling throughout the cell to reset its antioxidative capacity completely, thereby coping with the loss of a major NADH sink and enhancing resistance to ozone and *Tobacco mosaic virus* [20]. Schwarzländer *et al*. [89] studied the importance of mitochondria in oxidative stress and redox signaling by assessing the *in vivo* oxidation state of a redox-sensitive GFP targeted to *Arabidopsis* mitochondria. They demonstrated that mitochondria are highly sensitive to redox perturbation evoked by Cd, with their redox state recovering slower from an oxidative insult as compared to the cytosol or chloroplasts [89]. In addition, the mitochondrial ETC is also required to process excess reductants originating from the photosynthetic light reactions [21,33,77,90]. Overall, this suggests that mitochondria play a central role in perception as well as response signaling during the oxidative challenge in metal-exposed plants [17,89]. However, next to metal-induced ROS production and the thereby imposed (cellular) oxidative challenge, a direct link between metals and plant mitochondria is mediated by organic acids produced in the mitochondrial matrix [33]. These may be directly involved in the acclimation of plant cells to enhanced metal concentrations and will be discussed in the light of mitochondrial alternative respiration (Section 4.4).

### 4.1. Mitochondrial ROS-Induced Damage

Once ROS are formed in mitochondria of metal-stressed plants, they can either diffuse out of the mitochondria to mediate signaling functions or induce protein, lipid and DNA damage in the organelle itself [21] (Figure 2). Compared to nuclear DNA, the sensitivity of mitochondrial DNA to oxidative stress-induced damage is much higher due to the lack of chromatin organization and lower DNA repair activity [91]. Lipid peroxidation mediated by the interaction between ROS and membrane lipids can distort mitochondrial membrane integrity as shown by Panda *et al*. [43] in Al-stressed tobacco cells, thereby restricting ETC function. To detoxify lipid peroxidation products, plant mitochondria contain amongst other mechanisms a glutathione-S-transferase (GST) that was strongly upregulated in Cd-exposed plant cells, further supporting its critical role during metal stress (Table 1) [47].

Furthermore, plant mitochondrial proteins are susceptible to metal-catalyzed oxidation, leading to the irreversible formation of reactive carbonyl groups on amino acid side chains and hence reduced protein function [27]. Substantial evidence of both TCA cycle and photorespiration pathway proteins as important targets for ROS or lipid peroxidation products produced under environmental stresses such as drought, high light and metal exposure was summarized by Taylor *et al*. [92]. Bartoli *et al*. [93] have demonstrated a higher carbonyl accumulation in the wheat mitochondrial proteome as compared to other ROS-producing organelles such as chloroplasts and peroxisomes during well-irrigated and drought stress conditions. Mitochondrial protein carbonylation is rather selective, with not all proteins evenly susceptible to this process. This was demonstrated by Kristensen *et al*. [94], who have shown distinct subpopulations of the mitochondrial matrix proteome to be carbonylated after Cu and H_2_O_2_ treatment. Mitochondrial aconitase seems particularly prone to oxidative damage [63] as it is either carbonylated after *in vitro* oxidation with Cu and H_2_O_2_ [94] or fragmented after oxidative stress [55]. In addition, its abundance was decreased in Cd-exposed *Arabidopsis thaliana* cells [47].

### 4.2. The Role of Plant Mitochondria in Metal-Induced Programmed Cell Death

Although enhanced mitochondrial ROS levels may serve as monitors and signal the extent of environmental stress throughout plant cells, they may also lead to oxidative damage and programmed cell death (PCD) when mitochondrial and/or cellular antioxidative defense and repair systems are overwhelmed. Programmed cell death is an active and genetically controlled process essential for growth and development, as well as for adaptation to altered environmental conditions [95]. In animals, the essential role of mitochondria in the signaling pathway transducing specific signals into the execution of cell death is widely accepted [96]. A similar function was suggested for plant mitochondria [97], with H_2_O_2_ and other ROS as signals modulating plant PCD. The main event in plant PCD is the release of mitochondrial cytochrome c [98]. This was shown to occur in Al-stressed tobacco cells by Panda *et al*. [43]. In addition, ROS potentially derived from or interfering with mitochondrial functioning could be key mediators of metal-induced cell death as discussed in several research papers. Exposure to Zn induced cell death in rice roots, which could be mediated by ROS derived from the mitochondrial ETC [53]. Recently, Li and Xing [44] investigated possible mechanisms underlying Al-induced PCD in *Arabidopsis* mesophyll protoplasts using fluorescence techniques to monitor the *in vivo* behavior of plant mitochondria and caspase-3-like activity. After a quick ROS burst, mitochondrial swelling and loss of the transmembrane potential occurred prior to PCD. Application of AsA prior to Al-exposure slowed down but did not prevent these processes [44]. Mitochondrial O_2_°^−^ production—rather than NADPH oxidase-derived extracellular H_2_O_2_—was shown to be a key event in Cd-induced cell death in tobacco cells [48]. In addition, NO and ROS may be co-involved in Cd-mediated PCD as shown by De Michele *et al.* [41] and recently discussed by Arasimowicz-Jelonek *et al.* [42]. Although our insights are currently increasing (Table 1), more research is required to fully unravel the importance of plant mitochondria and the specific mediators involved during metal-induced PCD, which could be a regulated defense response to improve plant survival under metal stress conditions.

### 4.3. Mitochondrial Retrograde Signaling during Metal Stress in Plants

Depending on the intensity of the stressor, metals are able to induce mitochondrial damage and/or signaling outside the mitochondria. An altered organellar redox state generates signals that are transmitted to the nucleus in a process called retrograde signaling. This process occurs between mitochondria, chloroplasts and the nucleus and can be mediated by ROS or oxidative stress-induced secondary signals. Recently, Suzuki *et al*. [99] reviewed the intense relationship between mitochondria and chloroplasts in stress-induced redox signaling throughout the cell. Galvez-Valdivieso *et al*. [100] summarized the involvement of ROS in chloroplastic retrograde signaling, for which more data are available as compared to mitochondria. Due to their ability to signal across organellar membranes, ROS are considered as key components transducing signals between organelles. The dynamics and specificity of ROS-induced signaling are still questioned, but were recently reviewed by Mittler *et al*. [101]. They suggest ROS signaling to be a dynamic process occurring within cells between different organelles, as well as over long distances between different cells. In addition, ROS-induced oxidative damage to mitochondrial components may produce secondary signals. Møller and Kristensen [102] reviewed the potential of ROS-mediated protein oxidation in plant mitochondria as a stress indicator. Oxidatively damaged proteins such as those functioning in the TCA cycle and antioxidative defense can either be degraded by proteases or serve as an alarm signal to initiate plant responses at the cellular level [102,103].

To date, no specific components of any mitochondrial retrograde signaling pathway have been identified [99]. However, a mechanism of retrograde signaling is suggested to be involved in plant stress responses to excess Al as *AOX* transcription is increased [44,104]. Therefore, the involvement of retrograde mechanisms in oxidative stress and/or signaling induced by other metals is plausible and deserves further attention. Recently, Van Aken *et al*. [105] demonstrated that plant mitochondria respond to a wide variety of abiotic stresses (e.g., salt and heat), chemical inhibitors (e.g., rotenone) and hormones (e.g., abscisic and salicylic acid). They also offered a range of stress-responsive genes as potential targets to study novel mitochondrial retrograde signaling pathways.

The most intensively studied model for retrograde signaling between the mitochondrion and nucleus resulting in acclimation to stress conditions is AOX (Section 3.1, Figure 2). This enzyme could play a pivotal role during metal-induced signaling in plant mitochondria since AOX transcription, protein content and/or activity/capacity are commonly increased upon metal exposure (Table 1). Induction of AOX correlates with an enhanced tolerance to ozone and *Tobacco mosaic virus* in tobacco plants [20], potentially by altering organellar and/or cellular ROS levels [16] which could also be important during metal stress. Van Aken *et al*. [22] suggest that AOX is not only a target, but also a regulator of diverse stress responses in plants, mainly based on the results of studies using transgenic plants either with increased or reduced AOX content. The absence of AOX in *Arabidopsis* resulted in acute sensitivity to combined drought and light stress, confirming a role for AOX in determining the steady-state cellular redox balance [106]. This further suggests the possible involvement of AOX during acclimation to metal exposure in plants. *Arabidopsis* protoplasts lacking the gene coding for the major isoform *AOX1a* showed a dramatically decreased viability during Al exposure as compared to wildtype protoplasts [44]. Conversely, *AOX1a* overexpression enhanced Al tolerance, confirming the protective role of AOX against Al-mediated PCD [44]. The possible function of AOX as a mitochondrial “survival” protein was suggested by Robson and Vanlerberghe [107] in transgenic tobacco cells lacking AOX exposed to H_2_O_2_, salicylic acid and the protein phosphatase inhibitor cantharidin. It is hypothesized that the survival function of AOX is based on its ability to continuously suppress mitochondrial ROS generation. This further prevents oxidative damage that could otherwise evoke disturbed gene expression and favor PCD. In addition, the maintenance of respiration in stressful conditions by the alternative route also contributes to the hypothesis of AOX as a survival protein [107]. Vanlerberghe *et al*. [108] recently reviewed the postulated metabolic and physiological roles of the alternative respiratory pathway, with AOX possibly maintaining a homeostatic mitochondrial signal during stress conditions. Interestingly, the AsA biosynthesis capacity increased in isolated mitochondria of plants overexpressing AOX [109], suggesting a link between this energy-dissipating enzyme and mitochondrial and cellular antioxidative metabolism. Further research using transgenic plants exposed to different metals will contribute to our insights into the role of AOX in metal-induced (retrograde) signaling. However, other proteins and/or metabolites should also be studied to fully unravel the mechanisms involved in the acclimation of plants growing on metal-contaminated soils.

### 4.4. Metal Tolerance Mediated by Mitochondrial Organic Acids

In stress conditions compromising the phosphorylating cytochrome respiratory pathway, such as metal exposure, plant mitochondria use their alternative respiratory pathway to maintain the electron flux to O_2_. As discussed earlier, this may reduce mitochondrial ROS production by electron leakage from the system, thereby decreasing metal-induced oxidative stress at organellar and possibly cellular levels. In addition, the sustained electron flux allows a continuously operating glycolysis and TCA cycle, thereby ensuring a great metabolic flexibility under stress conditions [33]. This provides an alternative for the non-phosphorylating respiratory bypasses to ameliorate metal stress—next to their proposed role in modulating ROS production. Indeed, an increased TCA cycle flux results in the production of organic acids in the mitochondrial matrix. These metabolites form a direct link between plant mitochondria and acclimation to metal stress as they are associated with metal hyperaccumulation and tolerance in several plant species. Citrate, malate and oxalate have been suggested as key cellular ligands for Cd, Ni and Zn, mediating metal transport through the xylem and vacuolar sequestration of metal-ligand complexes (for reviews see [33,110,111] and references therein). In *Lycopersicon esulentum*, Cd-induced secretion of oxalate from root apices was found to be associated with Cd resistance [112]. In addition, both citrate and malate were shown to be secreted from root apices of plants exposed to Al, thereby establishing an Al tolerance mechanism [113]. Singh and Chauhan [114] also discussed the potential of organic acids to detoxify absorbed Al in the cytosol, followed by vacuolar sequestration as an internal tolerance mechanism exploited by several Al accumulating plant species. Interestingly, Gray *et al*. [115] have demonstrated an increased *AOX1* transcript level in tobacco cells incubated with organic acids such as citrate, malate and 2-oxoglutarate in a physiologically relevant concentration range. The authors postulate a plant mitochondrial retrograde signaling pathway for the regulation of AOX gene expression based on TCA cycle intermediates, which could function concomitantly with ROS signaling to the nucleus (Figure 2) [115].

## 5. Conclusions

Due to their central position in the cellular metabolism, the close relationship with chloroplasts and the integration of redox signals, plant mitochondria are main players in metal-induced cellular responses. In addition to being a target of cytosolar ROS, they also represent an important source of ROS production in conditions of metal stress. Depending on the intensity of the stressor, ROS are able to induce mitochondrial damage and/or signaling outside the mitochondria. The involvement of (mitochondrial) ROS in metal-induced PCD has been confirmed in several studies. Functioning as both a target and regulator of stress responses in plants, AOX is of major importance in the mitochondrial metabolism. Due to its ability to reduce mitochondrial ROS production, modulate PCD and TCA cycle activity, AOX is suggested to play a key role in metal-induced responses in plant mitochondria (Figure 2). Further research is needed to explore the role of this and other mitochondrial proteins in PCD and/or signaling-induced acclimation in more detail. Unraveling the role of mitochondria in metal-induced oxidative stress will ultimately contribute to the development and/or selection of crops with enhanced yield under suboptimal conditions such as metal exposure.

## Figures and Tables

**Table 1:** Exposure to excess metals has consequences for plant mitochondria at different levels. The effects of excess Al, Cd, Cr, Cu, Fe, Pb and Zn are shown and categorized based upon the experimental setup used (isolated mitochondria (A), cell cultures/protoplasts (B) or intact plants (C)). Metals impose a mitochondrial oxidative challenge characterized by an increased ROS production and altered antioxidative defense. This oxidative challenge is most likely the result of metal-induced ETC dysfunction at the level of the cytochrome pathway. Several metals activate the alternative respiratory pathway at different levels, but also induce mitochondrial damage *versus* signaling and defense (e.g., programmed cell death) responses. Metal-induced responses related to plant mitochondria are described schematically in the column “Observations”.

A. METAL-INDUCED RESPONSES IN ISOLATED MITOCHONDRIA						

Metal	Concentration	Exposure Time	Setup	Species	Observations	Ref.
**Al**	50 μM	18 h	Isolation after exposing cells	*N. tabacum*	↑ ROS production (O_2_°*^−^* and H_2_O_2_) ↓ O_2_ consumption ↓ ATP content ↓ cytochrome capacity ↓ AOX capacity opening of mitochondrial permeability transition pore cytochrome c release and nuclear fragmentation ~ PCD distorted mitochondrial membrane architecture	[43]
	0.1–0.5–1 mM	60 min	Exposure after isolation out of mesophyll protoplasts	*A. thaliana*	↑ ROS production (O_2_°^−^ and H_2_O_2_) ↓ complex I and III activity	[44]

**Cd**	10–30 μM	30 min	Exposure after isolation out of tubers	*S. tuberosum*	↑ ROS production (O_2_°^−^ and H_2_O_2_)	[36]
	5 mM	12 to 120 h	Isolation after exposing germinating seeds	*P. sativum*	↓ glutaredoxin, GR, GSH	[39]

**Cr**	20 or 200 μM	7 days	Isolation out of roots after exposing plants	*P. sativum*	↑ O_2_°^−^ lipid peroxidation of mitochondrial membranes altered SOD activity ↓ respiratory complex activity (IV most sensitive)	[31]

**Cu**	2–20–50 μM	6 days	Isolation after exposing cells	*A. pseudoplatanus*	↑ alternative respiratory pathway (KCN-resistant) ↑ AOX protein content	[30]

**Pb**	0.1–0.5 mM	Up to 3 days	Isolation out of roots after exposing plants	*P. sativum*	↑ H_2_O_2_ (mitochondria main site) ↑ AOX transcription and protein content	[45]
	0.5–1 mM	2 to 96 h	Isolation out of roots after exposing plants	*P. sativum*	↑ MnSOD activity ↑ alternative respiratory pathway (KCN-resistant) ↑ AOX protein content ↓ number of mitochondrial cristae	[46]

**Al**	25–50–75–100 μM	6 to 24 h	Cell culture	*N. tabacum*	↑ ROS production (O_2_°^−^ and H_2_O_2_) ↓ mitochondrial activity ↓ respiration (O_2_ uptake) ↓ ATP content	[32]
	0.5 mM	60 to 100 min	Protoplasts	*A. thaliana*	↑ ROS production (O_2_°^−^ and H_2_O_2_) ↑ *AOX1a* transcription ↓ mitochondrial transmembrane potential ↑ caspase-3-like protease activity ~ PCD disrupted mitochondrial ultrastructure	[44]

**Cd**	20 μM	5 h	Protoplasts	*A. thaliana*	↑ H_2_O_2_ in mitochondria prior to chloroplasts mitochondrial clustering and restricted movement	[37]
	0.5–2–5–20– 50–200 μM	24 h	Cell culture	*A. thaliana*	↑ MDHAR, peroxiredoxin	[47]
	100 or 150 μM	3 days	Cell culture	*A. thaliana*	↑ PCD	[41]
	3 mM	1 h	Cell culture	*N. tabacum*	↑ O_2_°^−^	[48]

**Cu**	2–20–50 μM	Up to 6 days	Cell culture	*A. pseudoplatanus*	↓ respiration (O_2_ uptake) ↑ alternative respiratory pathway (KCN-resistant) ↑ *AOX1* transcription	[30]

**Al**	5–10–15– 20 μM	4 to 24 h	Root apices	*P. sativum*	↓ respiration (O_2_ uptake) ↓ ATP content	[32]
	100 μM	1 to 48 h	Root tips	*T. aestivum*	↑ *MSD* transcription	[49]

**Cd**	30–60–100 μM	Up to 10 days	Roots and leaves	*H. distichum*	↓ respiration (O_2_ uptake) ↑ alternative respiratory pathway (SHAM)	[50]

**Cr**	2–5–10 mg/L	6 days	Leaves	*S. minima*	↑ AOX capacity (SHAM)	[51]

**Cu**	2 or 5 μM	24 h	Roots	*A. thaliana*	↓ *MSD1* transcription	[52]

**Fe**	100 μM	12 h	Root cutting exposure	*N. plumbaginifolia*	↑ respiration in leaves before (O_2_ uptake)	[29]

**Zn**	1–5–10– 25 mM	10 min to 9 h	Roots	*O. sativa*	mitochondrial ROS potentially involved in cell death	[53]

Abbreviations: AOX, alternative oxidase; GR, glutathione reductase; GSH, reduced glutathione; H_2_O_2_, hydrogen peroxide; KCN, potassium cyanide; MDHAR, monodehydroascorbate reductase; MnSOD, manganese superoxide dismutase; MSD1, manganese superoxide dismutase isoform 1; O_2_°*^−^*, superoxide; PCD, programmed cell death; SHAM, salicylhydroxamic acid; SOD, superoxide dismutase.
